# A novel Markov Blanket-based repeated-fishing strategy for capturing phenotype-related biomarkers in big omics data

**DOI:** 10.1186/s12863-016-0358-5

**Published:** 2016-03-09

**Authors:** Hongkai Li, Zhongshang Yuan, Jiadong Ji, Jing Xu, Tao Zhang, Xiaoshuai Zhang, Fuzhong Xue

**Affiliations:** Department of biostatistics, School of Public Health, Shandong University, Jinan City, Shandong Province P. R. China

**Keywords:** Big omics data, Markov Blanket-based repeated-fishing strategy (MBRFS), Phenotype-related biomarkers

## Abstract

**Background:**

We propose a novel Markov Blanket-based repeated-fishing strategy (MBRFS) in attempt to increase the power of existing Markov Blanket method (DASSO-MB) and maintain its advantages in omic data analysis.

**Results:**

Both simulation and real data analysis were conducted to assess its performances by comparing with other methods including *χ*^2^ test with Bonferroni and B-H adjustment, least absolute shrinkage and selection operator (LASSO) and DASSO-MB. A serious of simulation studies showed that the true discovery rate (TDR) of proposed MBRFS was always close to zero under null hypothesis (odds ratio = 1 for each SNPs) with excellent stability in all three scenarios of independent phenotype-related SNPs without linkage disequilibrium (LD) around them, correlated phenotype-related SNPs without LD around them, and phenotype-related SNPs with strong LD around them. As expected, under different odds ratio and minor allel frequency (MAFs), MBRFS always had the best performances in capturing the true phenotype-related biomarkers with higher matthews correlation coefficience (MCC) for all three scenarios above. More importantly, since proposed MBRFS using the repeated fishing strategy, it still captures more phenotype-related SNPs with minor effects when non-significant phenotype-related SNPs emerged under *χ*^2^ test after Bonferroni multiple correction. The various real omics data analysis, including GWAS data, DNA methylation data, gene expression data and metabolites data, indicated that the proposed MBRFS always detected relatively reasonable biomarkers.

**Conclusions:**

Our proposed MBRFS can exactly capture the true phenotype-related biomarkers with the reduction of false negative rate when the phenotype-related biomarkers are independent or correlated, as well as the circumstance that phenotype-related biomarkers are associated with non-phenotype-related ones.

**Electronic supplementary material:**

The online version of this article (doi:10.1186/s12863-016-0358-5) contains supplementary material, which is available to authorized users.

## Background

High-throughput-omic platforms, such as SNPS arrays, expression arrays and mass spectrometry, etc., have been commonly used in large scale population level systems biology or systems epidemiology study. These omic techniques have provided us the feasibility to accumulate a wealth of genetic, transcriptomic, proteomic and metabolomics data to study health and disease in breadth and depth at the human population level. Therefore, integrating various omic-metrics and environmental factors at population level will be crucial for developing effective diagnostic techniques, new drugs and intervention measures for disease. Among these, one key task is to map phenome (phenotype and various molecular phenotypes) onto genome, including mapping phenotype onto genome (GWAS), transcriptome onto genome (eQTL), proteome onto genome (pQTL), metabolome onto genome (mGWAS), metabolome onto epigenome (mEWAS), epigenome onto genome (meQTL), and phenome onto metabolome (MWAS) [1]. Nevertheless, how to hunt the phenome-related biomarkers has posed great challenge in such big omics data.

Currently, two strategies, statistical hypothesis tests and variable selection methods, can usually be adopted to handle the above mentioned problems. For the former, single marker-single phenotype hypothesis tests (e.g. *χ*^2^ test and t test) with *p* values adjusted for multiple comparisons by Bonferroni or B-H methods, have been customarily regarded as the universal criterion to claim the significance of each marker [2]. Obviously, these arbitrary correction methods inevitably increase the false negatives, for instance, phenotype-related biomarkers with *p* value larger than the Bonferroni cutoff of *χ*^2^ test will never be identified as positive ones. Moreover, the most commonly used Bonferroni adjustment will be less powerful if the high correlation existed between markers (e.g., linkage disequilibrium, LD, between SNPs), which can be ubiquitously encountered in big omics data analysis.

The second strategy is an alternative strategy mainly focusing on variable selection under machine learning framework. Various models have been successfully used to identify biomarkers in omics data, including bridge regression [3], least absolute shrinkage and selection operator (LASSO) [4], smoothly clipped absolute deviation (SCAD) [5], elastic net [6], adaptive lasso [7] and GWASelect [8]. However, few methods can be used to effectively distinguish the true phenotype-related biomarkers (e.g. SNPs) from its high correlated non-phenotype-related biomarkers (e.g. linkage disequilibrium with the phenotype-related SNPs in GWAS). Actually, capturing the phenotype-related biomarkers exactly will shorten the time required for further function verification and results in potential cost savings. One possible method to achieve this is to fully utilize the conditional independence property between the potential phenotype-related biomarkers and their related ones in the framework of directed acyclic graph (DAG). Theoretically, as a tool based on Markov independent property, the Markov Blanket (MB) [9] aimed to search a minimal biomarker set given which all other biomarkers are probabilistically independent with the specific phenotype. Thus in practice, Markov Blanket seems to be able to effectively hunt the true phenotype-related biomarkers (e.g. SNPs) rather than their high correlated non-phenotype-related biomarkers.

**Fig. 1:**
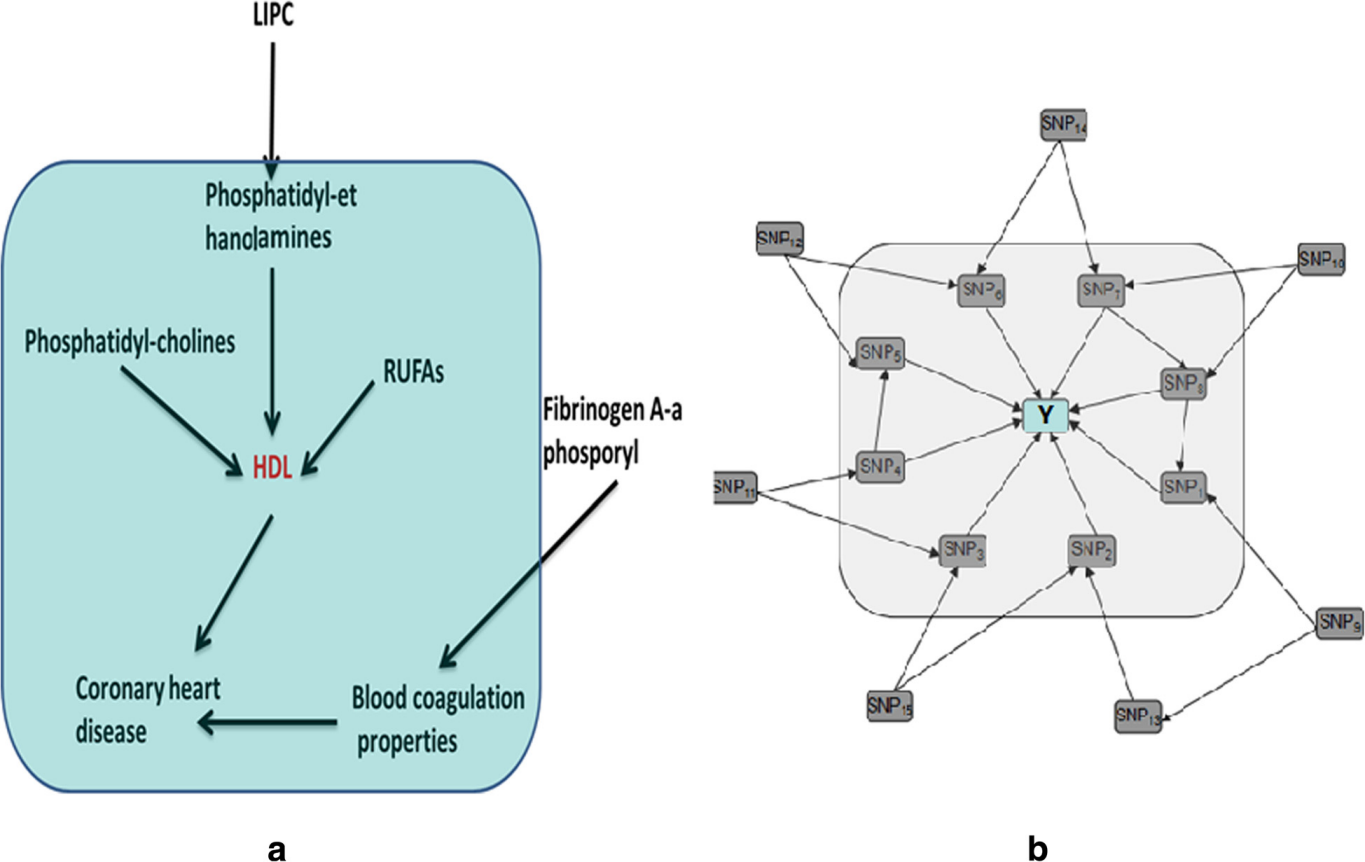
The Markov Blanket of high density lipoprotein (HDL) and Y. **a** The Markov Blanket of HDL denoted as five factors denoted with blue box (i.e. Phosphatidyl-ethanolamines, RUFAs, Phosphatidyl-cholines, Coronary heart disease and Blood coagulation properties); **b** The Markov Blanket of Y denoted as SNPs (i.e. SNP1 ~ SNP8) around with shadow box

Recently, two MB-based approaches proposed by Bing Han et al*.* had been used to detect gene-gene interaction in GWAS. The authors claimed that their algorithms outperformed other computational methods for detecting gene-gene interaction. However, when their methods (DASSO-MB [10] and FEPI-MB [11]) were applied to detect the SNPs associated with Leprosy containing 490,000 SNPs in our case–control GWAS datasets (706 case and 514 controls), only few significant SNPs (2 for DASSO-MB and 3 for FEPI-MB respectively) were identified at nominal level of 0.05. Similarly, the authors reported only 2 SNPs for both above methods in analyzing the GWAS datasets of Age-related Macular Degeneration (AMD) with 116,204 SNPs (96 cases and 50 controls). Hence it seems to be unreasonable to apply DASSO-MB and FEPI-MB to interpret the genetic mechanism for multiple-factorial complex diseases, which commonly caused by numerical SNPs distributing on the whole genome. In previous studies, even though quite a few genetic variants have been successfully identified by GWAS, they still accounted for a small proportion of the total heritability for complex diseases. Therefore, developing novel data analysis methods are highly desirable to detect more phenotype (or disease)-related biomarkers, meaning explaining more heritability. Further insight into DASSO-MB [10] and FEPI-MB [11] told us that they would lose power when numerous phenotype-related SNPs existed on the whole genome due to the drawbacks of the algorithm strategy, much worse, both algorithms might not work when a phenotype-related SNP located in a high LD block. This is because the conditional independence test relied on *G*^2^ statistic, which needed to stratify the conditioned variables (e.g. SNPs) already selected in Markov Blanket (MB). As shown in Fig. 1b below, suppose ten SNPs under additive model are associated with disease in real world genome, the number of stratification needed will reach 3^9^ = 19683 with totally 6 × 19683 = 118098 cells if we expect to capture all the ten SNPs exactly. Then, the sample size required for such enormous cells will be formidable and unimaginable.

We, therefore, proposed a novel Markov Blanket-based repeated-fishing strategy (MBRFS) for capturing phenotype-related biomarkers in big omics data. Imagining the phenotype-related biomarkers (e.g. phenotype-related SNPs) mixed in the high dimensional omics variable pool (e.g. tag SNPs on the whole genome) with a given proportion, the idea of our strategies stemmed from which we can use the MB algorithm (i.e. fishing net) to capture the phenotype-related SNPs (i.e. fish) repeatedly from omics variable pool. As the biomarker set captured in MB (i.e. fishing net) each time were immediately taken off from the MB (i.e. being emptied), the next re-constructed MB always maintained a few new phenotype-related biomarkers to make sure the empty cells were not too many. Thus, the *G*^2^ statistic (i.e. likelihood ratio chi-square statistic) or other methods (e.g. regression model) for testing conditional independence property could always hold the relative high power under given sample size. Taking the simplest omics data of GWAS as an example, simulations were conducted to assess its performances with true discovery rate (TDR), false discovery rate (FDR) and matthews correlation coefficience (MCC) [12] and compared with the performances of commonly used statistical hypothesis tests method (*χ*^2^ test with Bonferroni or B-H adjustment), variable selection method (LASSO), as well as existing MB-based methods (DASSO-MB). For case study, various real omic data, including GWAS data for Leprosy, DNA methylation & gene expression data for breast cancer, and metabolomic data for schizophrenia were analyzed.

## Methods

### Markov Blanket-based repeated-fishing strategy (MBRFS)

The Markov Blanket of a target variable of Y, MB(Y), was defined as a minimal set given which all other variables were independent with Y, i.e. all other variables are probabilistically independent of the variable Y conditioned on the MB of variable Y [9]. Figure 1a shows a commonly used MB example of high density lipoprotein (HDL), referred to given Phosphatidyl-ethanolamines, RUFAs, Phosphatidyl-cholines, Coronary heart disease and Blood coagulation properties, the HDL is conditionally independent of LIPC and Fitbrinogen A-a phosporyl. Specifically, for the MB under GWAS data, from the perspective of causality between genetic variation and phenotype, we usually suppose phenotype (i.e. complex diseases Y) never causes genetic variation (i.e. SNPs). Thus, MB with 10 phenotype-related SNPs can be expressed as Fig. 1b.

In previous studies, many MB algorithms had been proposed, including KS algorithm [13], GS algorithm [14], IAMB [15], MMMB [9] and HITON-MB [16], DASSO-MB [10], FEPI-MB [11] etc. Among them, DASSO-MB and its updated version of FEPI-MB had lower false positive by adding a backward phase after each step of selecting a variable in the forward phase. However, as DASSO-MB attempted to make the size of MB(Y) as small as possible, it would inevitably increase false negative rate, especially, numerous phenotype-related biomarkers existing in high dimensional omics data, which is much common in omics study of complex diseases. There are two types of phases in DASSO-MB containing forward phase and backward phase. In each loop of the forward phase, if one variable with a maximal G2 score conditioned on MB(Y) is dependent on target variable Y, it will be putted into MB(Y). This admission operation is followed by a backward phase to remove false positives SNPs by performing conditional independence tests. If no more variable will be added into MB(Y) in the forward phase, they will enter the final backward phase to remove variables that do not belong to MB(Y). More details about DASSO-MB can be found in [10]. In this paper, our proposed MBRFS not only holds the advantage of DASSO-MB on reducing false positive rate, but decreases the false negative rate by repeated-fishing strategy.

**Fig. 2:**
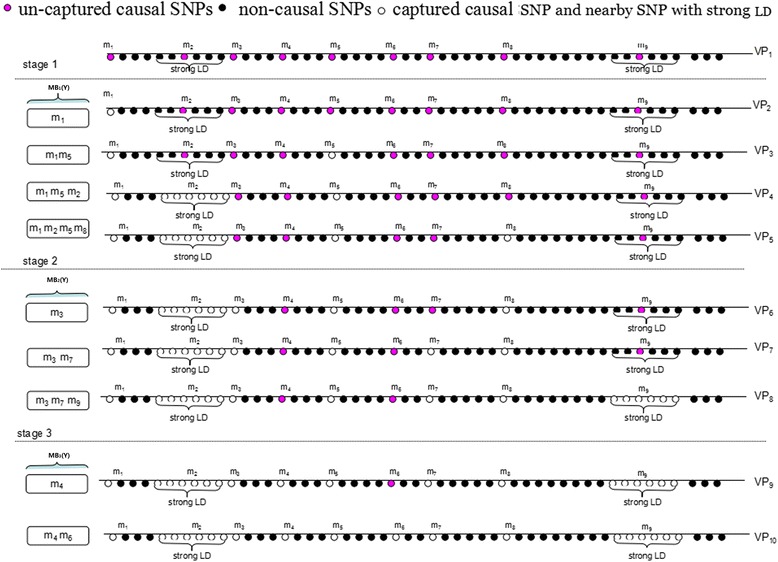
Variable selection process of MBRFS. VP1-VP10 was the constantly updated variable pools, MB1(Y), MB2(Y), MB3(Y) maked up the final MB set of MBRFS

In order to increase the power of DASSO-MB and maintain its advantages, we modified the algorithm in three aspects. Firstly, from original high dimensional omics variables, the initially screening procedure by single statistical test (e.g. *χ*^2^ test for categorical phenotype, etc.) at the nominal level 0.05 was performed before MB search algorithm. This strategy not only improved the computation speed, but detected the marginal association between biomarkers and phenotype as many as possible for further conditional independent test in MB algorithm. Secondly, in order to reduce the number of empty cells of the hierarchical contingency table in *G*^2^ test when selecting a new biomarker into MB, we relaxed the conditional independent criterion by just using one order combination of biomarkers already within MB as condition. For example, supposing V_1_, V_2_, V_3_ had been in MB(Y) , we selected the outside new variable V_4_ into MB(Y) if {*V*_4_ ⊥ *Y*|*V*_1_} ∩ {*V*_4_ ⊥ *Y*|*V*_2_} ∩ {*V*_4_ ⊥ *Y*|*V*_3_}. In the next step, the proposed repeated-fishing strategy (MBRFS) was further used to resolve problem of too many empty cells in hierarchical contingency table to maintain the power of *G*^2^ test. Figure 2 showed the framework of our proposed MBRFS algorithm.

- Initially screen the biomarkers at the nominal level 0.05 from original high dimensional omics variables by single marker statistical test (e.g. *χ*^2^ test for categorical phenotype, etc.) to establish candidate variable pool.
- Capture the first subset (i.e. m_1_, m_2_, m_5_, m_8_) of phenotype-related biomarkers using the modified DASSO-MB algorithm. As shown in variable pool 1 (VP_1_), m_1_ was selected into the MB_1_(Y) with the minimal *p* value, then VP_1_ was updated to be VP_2_. Given VP_2_, select the biomarker m_5_ into MB_1_(Y) hence updated VP_2_ to be VP_3_. Moreover, given VP_3_, since m_2_ was located in the high correlated region, once it was captured into MB_1_(Y), other biomarkers in this region were removed under given criteria. For example, if a SNP in high LD block was captured by MB algorithm, then other SNPs, which have *r*^2^ > 0.05 with this captured SNP and within 20 SNPs around it, were removed from candidate variable pool [8]. In addition, VP_3_ was updated toVP_4_ while m_8_ being selected into MB_1_(Y) when no other biomarkers entered. Finally MB_1_(Y) was constructed with m_1_, m_2_, m_5_ and m_8_, and VP_4_ was updated to VP_5_.
- Repeat the process in step (b), we obtain MB_2_(Y) with m_3,_ m_7_ and m_9_, the variable pool was updated to VP_8_.
- MB_3_(Y) with m_4_ and m_6_ as well as VP_10_ can also be obtained. For details, see stage 3 in Fig. 2.
- The next MB(Y) was continuously constructed from VP_10_ until only two biomarkers were selected in the blanket.

Under the framework of MBRFS, we developed several MB algorithms by modifying DASSO-MB algorithm which are suitable to be applied to different omics data (Additional file 1: Figure S1). Suppose *X* = {*x*_1_, *x*_2_^*m*^, *x*_3_, …, *x*_*i*_^*m*^, …, *x*_*p*_} denoted the *p* biomarkers in omics variable pool, where superscript *m* indicated that the specific biomarkers (e.g. *x*_2_^*m*^, etc.) were related (or causal) to the phenotype (*Y*). We defined the MB of target phenotype as *MB*(*Y*) which was a minimal set subjected to *V* ⊥ *Y*|*MB*(*Y*) for all *V* ∈ *X* − *MB*(*Y*). As shown in Fig. 1b, *MB*(*Y*) is a set of gray-filled nodes {SNP_1_, SNP_2_, SNP_3_, SNP_4_, SNP_5_, SNP_6_, SNP_7_, SNP_8_}, and variables SNP_9_-SNP_15_ are independent of *Y* conditioned on *MB*(*Y*). In our proposed MBRFS, the solution for conditional independent tests in MB algorithms is given as follows:

- For catagorical biomarkers and phenotype, *G*^2^ statistic, ${G}^2=2{\displaystyle \sum_{i=1}^n{O}_i \ln \left(\frac{O_i}{E_i}\right)},$ would be used, it follows an asymptotical *χ*^2^ distribution with degree of freedom $df=\left(\mathrm{Cat}\left(\mathrm{A}\right)-1\right)\times \left(\mathrm{Cat}\left(\mathrm{B}\right)-1\right)\times {\displaystyle \prod_{i=1}^n Cat\left({\mathrm{C}}_i\right)},$ where Cat(X) was the number of categories of the variable X and n was the number of variables in C.
- For binary phenotype and quantitative biomarkers (e.g. MWAS, etc.), the conditional independent property was tested by logistic regression model, for example, log *it*(P(Y|*x*_2_^*m*^, *x*_3_)) = *α*_0_ + *β*_2_*x*_2_^*m*^ + *β*_3_*x*_3_ was used to detect whether *x*_3_ was independent of *Y* given *x*_2_^*m*^ based on the significance of *β*_3_.
- For biomarkers with quantitative phenotype, (e.g. mGWAS, mEWAS, pQTL, eQTL, and meQTL, etc.), their dependent variables (*Y*) were continuous. Therefore, ordinary linear regression model, e.g. *E*(Y|*x*_2_^*m*^, *x*_3_) = *α*_0_ + *β*_2_*x*_2_^*m*^ + *β*_3_*x*_3_, was adopted in conditional independent test.

### Simulation

Since GWAS is well-known and simplest omics study, a series of GWAS-based simulation were conducted to evaluate the performances of the proposed MBRFS, and compared it with machine learning methods (i.e. LASSO) [4], statistical Armitage trend *χ*^2^ test with Bonferroni [17] & B-H adjustment [18], as well as existing DASSO-MB [10] methods.

For whole genome data, considering various linkage disequilibrium (LD) patterns and Minor allele frequency (MAF) variation, 22 genome regions (each with 50 SNPs) were selected from 22 autosomal chromosomes respectively. Then a further merged mimic genome region with 1100 SNPs was created for conducting our simulation (Additional file 2: Figure S2). This simulated region was the miniature of whole genome, and covered the characteristics of the real world genome data. Software gs2.0 [19] was used to generate the genotype data with 100,000 individuals based on HapMap phase III CEU data (http://hapmap.ncbi.nlm.nih.gov/). The genotype data was coded by the additive genetic model.

Four simulation scenarios were designed for assessing the performances together with three commonly used indexes: true discovery rate (TDR), false discovery rate (FDR), and matthews correlation coefficience (MCC) [12] under different combinations of LD patterns, sample size, MAFs, effect size (OR), and degree of correlation between phenotype-related SNPs. In particular, MCC was a trade-off between false positive and false negative for comprehensively assessing the performances of different compared methods (see Additional file 3).

In simulation scenario 1, 8 independent phenotype-related SNPs were generated by logistic model (for details, see Additional file 4), then they were randomly inserted into 8 different chromosomes respectively in the simulated regions (Additional file 2: Figure S2). This idea stems from the gain-of-function technique which is usually taken to study the function of a gene [20, 21] so that it gets rid of the influence of LD completely.

For scenario 2, since phenotype-related genes (or SNPs) usually correlated within pathways or networks, a correlated pattern of the 8 phenotype-related SNPs was created by logistic model with correlation coefficient 0.1 between them (see supplement 2). Then the generated SNPs were also inserted into 8 different chromosomes respectively in the simulated regions following the same procedure of scenario 1 for evaluating the performances of our proposed MBRFS under correlated phenotype-related SNPs with no LD.

In scenario 3, 8 phenotype-related SNPs were randomly selected within 8 different simulated regions from different chromosomes respectively with various MAFs and LD patterns. The association between phenotype and selected SNPs was created by logistic model. Specifically, both true positive cluster (TPC) and false positive cluster (FPC) were defined in this scenario. Namely, if a captured SNP was no more than 20 SNPs away from a true phenotype-related SNP and had *r*^2^ > 0.05 with the same phenotype-related SNP, then we also regarded it as a true positive while for the situation of more than one SNP satisfied above conditions, we treated them only as one TPC. Simultaneously, the remaining captured SNPs were recognized as false positives; similarly, if the false positive SNPs were no more than 10 SNPs apart each other (i.e. within 100 kb in distance), they were counted as only one FPC, this criteria had been widely used to define TPC and FPC when the phenotype-related SNPs located in high LD regions [12]. Finally, the TDR, FDR and MCC were further calculated using TPC and FPC rather than individual SNPs.

For scenario 4, we selected three simulated regions with high (most pair of SNPs with *r*^2^ > 0.8), modest (with 0.2 < *r*^2^ < 0.8) and low LD (with *r*^2^ < 0.3) structure, each region contained 50 SNPs. We randomly chose one phenotype-related SNPs with OR = 1.5 in each simulation region, and performed 1000 simulations to assess the accuracy of compared methods using discovery rate of each SNPs, including the phenotype-related SNPs and every non-phenotype-related SNPs. For jth SNP, its discovery rate was defined as $\frac{\mathrm{the}\kern0.3em  number\kern0.2em  of\kern0.2em  dis\operatorname{cov} eried\kern0.2em SN{P}_j}{1000}\times 100\%$ where j = 1, 2, ⋯, 50.

For data analysis in each simulation scenario, the phenotype-related SNPs were retained in the generated data rather than discarded. The number of replications was set to be 1000 for each simulation scenario. All simulation studies were conducted using software R from CRAN (http://cran.r-project.org/).

Four simulation schemes in each simulation scenario using logistic regression model with case–control design were carried out respectively, including 1) to explore the stability of each method (MBRFS, *χ*^2^ test with Bonferroni and B-H adjustment, LASSO and DASSO-MB) under null hypothesis, namely the OR values of 8 SNPs were set to be 1 with MAFs = 0.3 under sample sizes *N* = 2000 (i.e. scheme 1); 2) to detect the TDR trend when effect size increased, OR for the 8 phenotype-related SNPs were set to be 1.1, 1.2, 1.3, 1.4, 1.5, 1.6, 1.7 and 1.8 successively, given MAFs = 0.3 & sample sizes 2000 (i.e. scheme 2); 3) various MAFs (from 0.05 to 0.5) were set, given OR = 1.3 & sample sizes 2000 (i.e. scheme 3); and 4) all 8 phenotype-related SNPs with non-significance by *χ*^2^ test after Bonferroni multiple correction were set, under different MAFs, OR and sample size. In this situation, we evaluated the performances of our proposed MBRFS in aspect of reducing false negative, and compared it with LASSO, DASSO-MB.

### Application

The publically available GWAS data, DNA methylation, gene expression and metabolomics data were analyzed using above five methods simultaneously, so as to confirm the advantages of the proposed MBRFS in practice. Furen Zhang et. al [22] reported a two-stage GWAS of Han Chinese located in eastern China, 93 SNPs most strongly associated with Leprosy were detected initially with 706 patients from Shandong Province and 1225 controls from several other provinces. After external validation in three independent samples with a total of 3254 patients and 5955 controls, 16 Leprosy-associated SNPs were finally identified. To compare the performances of our proposed MBRFS with Bonferroni adjustment after ATT test, B-H adjustment after ATT test, LASSO and DASSO-MB, the original GWAS data of Leprosy with a total of 491,883 SNPs from 706 case and 514 controls were analyzed using above compared five methods. Specifically, all of the sample (706 case and 514 controls) were only selected from Shandong Province to avoid the population heterogeneity, and to assess the performances of our proposed MBRFS to capture the phenotype-related SNPs under relatively smaller sample size. The DNA methylation and gene expression data of two subtypes of breast cancer (20 Infiltrating Ductal carcinoma patients and 22 Infiltrating Lobular carcinoma patients) were downloaded from http://cancergenome.nih.gov/. [23] The metabolomics data of schizophrenia contains 1723 metabolites with 58 cases and 71 controls from Shandong province in China. Logistic regression was used to test the conditional independent property for above DNA methylation, gene expression and metabolites data. By these various real data analysis, we evaluated whether our proposed MBRFS could be extended to the case with binary phenotype and quantitative biomarkers. Patient consent we obtained was written and it was informed. The study was approved by the Medical Ethical Committee of Qilu Hospital, Shandong University, China.

## Results

### Simulation studies

Table 1 showed the performances (overall TDR, FDR and MCC) of five methods above applied to four schemes in scenario 1, in which 8 independent phenotype-related SNPs randomly insert into the 8 different simulated regions respectively. In this scenario, we expected to examine the performances in the absence of the influence of LD, though it hardly existed in real world. It indicated that MBRFS had highest overall TDR with acceptable FDR in scheme 2 (i.e. the OR of 8 phenotype-related SNPs were set from 1.1 to 1.8) and in scheme 3 (i.e. the MAFs of 8 phenotype-related SNPs were set from 0.05 to 0.5). In scheme 4 (i.e. the OR and MAFs of 8 phenotype-related SNPs were set identically to 1.2 and 0.3 respectively), it showed that MBRFS and LASSO had relative higher overall TDR, while LASSO emerged highest FDR though its overall TDR seemed a little higher than the proposed MBRFS. Furthermore, MBRFS had highest MCC value in all three schemes when using MCC for assessing their performances.

**Table 1:** The overall TDR, FDR and MCC of three schemes of scenario 1 with 1000 cases and 1000 controls in each scheme

Methods	Scheme 2			Scheme 3			Scheme 4		
	TDR_overall_	FDR	MCC	TDR_overall_	FDR	MCC	TDR_overall_	FDR	MCC
Bonferroni	0.65	0.00	0.80	0.33	0.01	0.57	0.14	0.10	0.35
B-H	0.71	0.03	0.82	0.38	0.03	0.61	0.18	0.10	0.40
LASSO	0.52	0.00	0.72	0.75	0.17	0.78	0.76	0.44	0.65
DASSO-MB	0.52	0.01	0.72	0.43	0.11	0.65	0.24	0.28	0.41
MBRFS	0.80	0.14	0.83	0.78	0.16	0.81	0.58	0.20	0.68

Bonferroni and B-H is the multiply correction methods after *χ* ^2^ tests

*TDR* true discovery rate

*FDR* false discovery rate

*MCC* matthews correlation coefficience

**Fig. 3:**
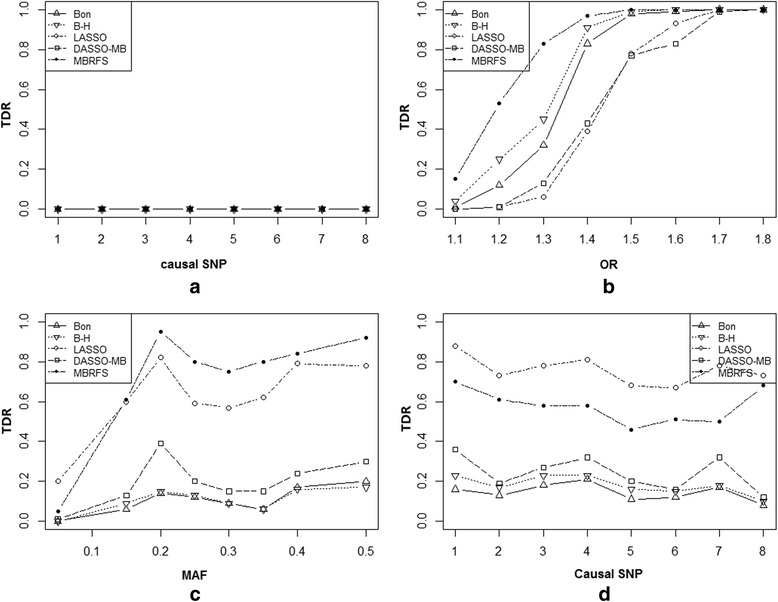
The performances of 5 methods including MBRFS, Bonferroni and B-H adjustment, LASSO, DASSO-MB in scenario 1. **a**-**d** are the results of four schemes of 5 methods in scenario 1

Figure 3 illustrated the TDR of compared methods for each phenotype-related SNP in scenario 1. Figure 3a presented the TDR under null hypothesis (i.e. OR = 1 for each SNPs) in scheme 1. As expected, the TDR of each method for each SNP was very close to zero. In scheme 2, the TDR of each method increased as OR went up, while the proposed MBRFS had the best performance (Fig. 3b). Generally, similar trend was also observed as MAFs of the phenotype-related SNPs gradually approximated to 0.5 in scheme 3 (Fig. 3c). Specifically, in scheme 4, both LASSO and MBRFS had stronger ability to capture the phenotype-related SNPs even with minor effect, which had been commonly missed by *χ*^2^ test with Bonferroni and B-H adjustment (Fig. 3d). It seemed that these two methods were able to reduce the false negative rate, however, LASSO had always kept higher FDR than MBRFS.

Table 2 showed the overall TDR and FDR of 5 methods in scenario 2 under the same three schemes as in scenario 1. Through randomly inserting 8 correlated phenotype-related SNPs into 8 different simulated regions, we aimed to evaluate the performances of 5 methods mentioned above when phenotype-related SNPs were correlated while no LD with other SNPs in the same region. It indicated that MBRFS and *χ*^2^ test with B-H adjustment had relatively higher overall TDR with acceptable FDR in scheme 2 and in scheme 3. While in scheme 4, it showed that MBRFS had the highest overall TDR, though its FDR seemed to be a little higher. Again, MBRFS had also highest MCC in the scheme 3 and 4.

**Table 2:** The overall TDR, FDR and MCC of three schemes of scenario 2 with 1000 cases and 1000 controls in each scheme

Methods	Scheme 2			Scheme 3			Scheme 4		
	TDR_overall_	FDR	MCC	TDR_overall_	FDR	MCC	TDR_overall_	FDR	MCC
Bonferroni	0.90	0.00	0.94	0.75	0.01	0.86	0.57	0.01	0.75
B-H	0.94	0.02	0.95	0.84	0.04	0.89	0.74	0.03	0.84
LASSO	0.51	0.00	0.71	0.78	0.01	0.88	0.82	0.09	0.86
DASSO-MB	0.54	0.00	0.71	0.45	0.07	0.66	0.36	0.11	0.56
MBRFS	0.98	0.12	0.93	0.92	0.14	0.89	0.96	0.13	0.91

Bonferroni and B-H is the multiply correction methods after *χ* ^2^ tests

*TDR* true discovery rate

*FDR* false discovery rate

*MCC* matthews correlation coefficience

**Fig. 4:**
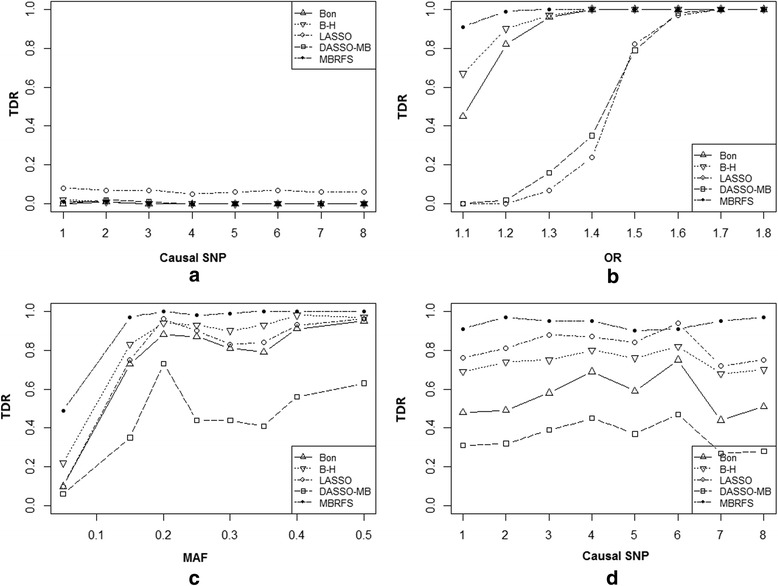
The performances of 5 methods including MBRFS, Bonferroni and B-H adjustment, LASSO, DASSO-MB in scenario 2. **a**-**d** are the results of four schemes of 5 methods in scenario 2

The TDR of 5 methods for each phenotype-related SNP in scenario 2 was presented in Fig. 4. Of which, Fig. 4a revealed that the TDR for each SNP of all methods were quite close to zero except LASSO in scheme 1. Similar phenomenon could be found in scheme 2–3 (Fig. 4b-c) as that in scenario 1. Figure 4d showed that MBRFS always held highest TDR than other methods in scheme 4.

What shown in Table 3 was the performances (overall TDR, FDR and MCC) of the above five methods applied to three schemes in scenario 3, in which the phenotype-related SNPs usually had various LD structures with its neighbor ones. In this scenario, we expected to examine their performances in the real world. It indicated that MBRFS had highest MCC (higher TDR and lower FDR) in scheme 2 (i.e. the OR of 8 phenotype-related SNPs were set from 1.1 to 1.8) and in scheme 3 (i.e. the MAFs of 8 phenotype-related SNPs were set from 0.05 to 0.5). In the scheme 4 (i.e. the OR and MAFs of 8 phenotype-related SNPs were set identically to 1.2 and 0.3 respectively), it showed that MBRFS and LASSO had relative higher overall MCC, while LASSO emerged highest FDR though its overall TDR was little higher than the proposed MBRFS. As emerged in above scenario 1 and 2, MBRFS still had highest MCC value in all three schemes. In addition, under different sample sizes we further compared the performances of five methods with fixed MAFs (0.3) and OR from 1.1 to 1.8 successively for the 8 phenotype-related SNPs. It suggested that our proposed MBRFS always had higher overall TDR. It was worth to note that the DASSO-MB did not work when sample size was relatively larger (more than 2000 in our simulation) due to its too many empty cells in conditional independent *G*^2^ test (Additional file 5: Figure S3).

**Table 3:** The overall TDR, FDR and MCC of three schemes of scenario 3 with 1000 cases and 1000 controls in each scheme

Methods	Scheme 2			Scheme 3			Scheme 4		
	TDR	FDR	MCC	TDR	FDR	MCC	TDR	FDR	MCC
Bonferroni	0.62	0.04	0.77	0.11	0.20	0.30	0.12	0.17	0.31
B-H	0.78	0.32	0.73	0.21	0.37	0.36	0.22	0.17	0.43
LASSO	0.56	0.01	0.74	0.64	0.57	0.52	0.65	0.55	0.54
DASSO-MB	0.43	0.01	0.65	0.18	0.43	0.32	0.23	0.30	0.40
MBRFS	0.74	0.11	0.81	0.66	0.20	0.72	0.51	0.21	0.63

Bonferroni and B-H is the multiply correction methods after *χ* ^2^ tests

*TDR* true discovery rate

*FDR* false discovery rate

*MCC* matthews correlation coefficience

**Fig. 5:**
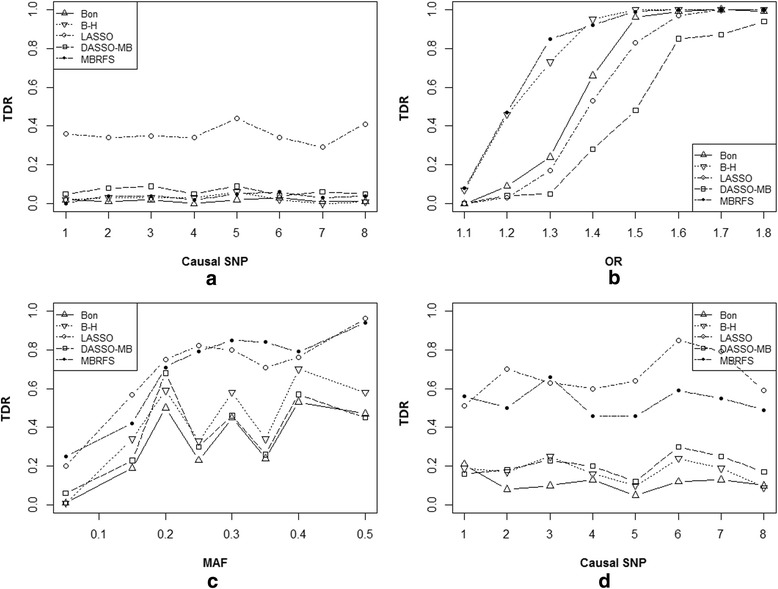
The performances of 5 methods including MBRFS, Bonferroni and B-H adjustment, LASSO, DASSO-MB in scenario 3. **a**-**d** are the results of four schemes of 5 methods in scenario 3

Figure 5 illustrated the TDR of compared methods for each phenotype-related SNP in scenario 3. What shown in Fig. 5a were the TDR under null hypothesis (OR = 1 for each SNPs) in scheme 1. As expected, the TDR of each method for each SNP were very close to zero except LASSO. In scheme 2, the TDR of each method increased as OR values went up, while the proposed MBRFS had the best performances (Fig. 5b). Generally, similar trend was also observed as MAFs of the phenotype-related SNPs gradually approximated to 0.5 in scheme 3 (Fig. 5c). Specifically, both LASSO and MBRFS had stronger ability to capture the phenotype-related SNPs even with minor effect, which had been commonly missed by *χ*^2^ test with Bonferroni and B-H adjustment methods in scheme 4 (Fig. 5d). It seemed that these two methods were able to reduce the false negative rate, however, LASSO had always kept higher FDR than MBRFS.

**Fig. 6:**
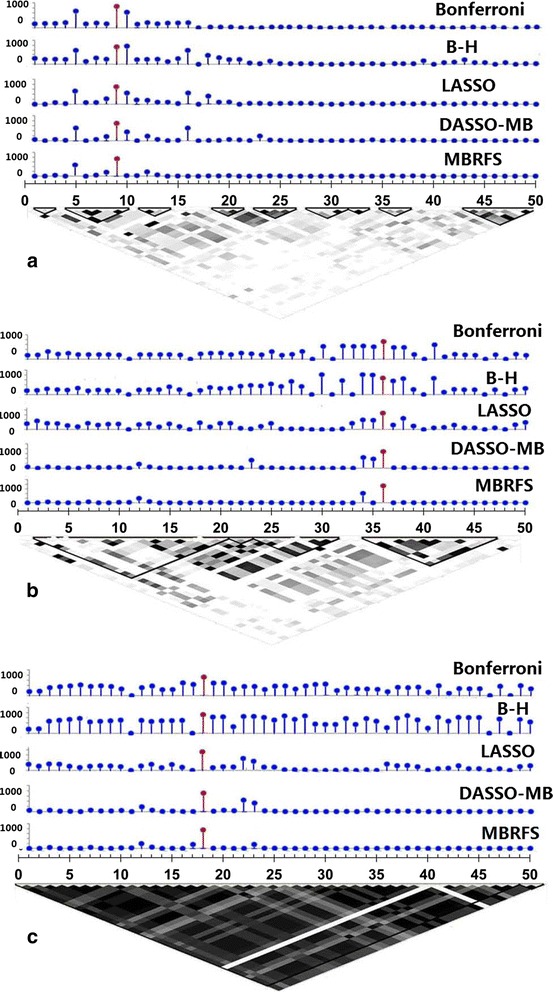
The performances of 5 methods including MBRFS, Bonferroni and B-H adjustment, LASSO, DASSO-MB in scenario 4. **a**-**c** illustrated the discovery rate of positive biomarkers in varied LD regions

For simulation scenario 4, we found that our proposed MBRFS could exactly identify the phenotype-related SNPs in the low, modest and higher LD region, while other methods, especially *χ*^2^ test with Bonferroni and B-H adjustment, selected more non-phenotype-related SNPs simultaneously (Fig. 6).

### Real data analys

Additional file 6: Table S1 shows the 18 positive clusters identified by our proposed method MBRFS for Leprosy GWAS data. Specifically, a positive SNPs clusters were defined as a set of SNPs meeting the following criteria: a) SNP located in the region with no more than 20 SNPs away from the positive SNP detected by MBRFS at the nominal level 0.01; b) all the SNPs in this region had *r*^2^ > 0.05 with the positive SNP and were statistically significant in the initial screening at the nominal level 0.01. Under this criteria, among the 18 positive SNPs clusters, 13 external validated SNPs in the two stage GWAS of Leprosy [22] were re-identified as Leprosy related SNPs by our proposed MBRFS (see Additional files 6 and 7: Table S1 and Figure S5). As expected, 8 negative SNPs under criteria of Bonferroni adjustment after ATT test were still detected by our proposed MBRFS (see Additional file 8: Figure S4). However, under the same criteria of positive SNPs clusters, only 7 external validated SNPs were re-identified as positive SNPs by B-H adjustment after ATT test, followed 5 by Bonferroni adjustment after ATT test, 3 by LASSO and 1 by DASSO-MB (see Additional files 6 and 7: Table S2 and Figure S5). With regard to computation time for analyzing the Leprosy GWAS data with a total of 491,883 SNPs using the same multiprocessor and multithreading computational cluster, the DASSO-MB had the longest computational time (2607 s), followed by MBRFS (1978 s), LASSO (804 s), Bonferroni adjustment after ATT test (301 s), and B-H adjustment after ATT test (302 s). This indicated that the computer burden was acceptable for our proposed method MBRFS in the real-world omics data analysis, though it took longer time than other three methods.

For DNA methylation and gene expression data on breast cancer, the results by the MBRFS and its compared methods were shown in Additional file 6: Table S3 and S4. This indicated that MBRFS detected relative reasonable genes, including CCDC91 [24], SCN4B.1 [25], C3orf10 [26] from DNA methylation data and NRXN1 [27], FAM71C [28], ZNF8 [29], FGL1 [30], ZNF438 [31], PTP4A3 [32], INCENP [33], SCTR [34], CYorf15A [35] from gene expression data, while other methods found too many genes to explain reasonably. For the schizophrenia metabolomics dataset, MBRFS identified three verified metabolites, including carnitine, pphingosine, stearamide [36], while LASSO method found fewer verified metabolites. The *p* value from logistic regression with Bonferroni and B-H adjustment found four verified metabolites, but they detected too many false positive ones (Additional file 6: Table S5). Of note, DASSO-MB method can only deal with catagorical biomarkers and catagorical phenotype and thus limits its application in above DNA methylation data, gene expression data, and metabolomics data.

## Discussion

In this paper, we proposed the novel Markov Blanket-based repeated-fishing strategy (MBRFS) for capturing phenotype-related biomarkers in big omics data. Simulations studies indicated that MBRFS generally outperformed other commonly used methods (*χ*^2^ test with Bonferroni and B-H adjustment, LASSO and DASSO-MB) under all three scenarios: independent phenotype-related SNPs without LD around them (Table 1 and Fig. 3), correlated phenotype-related SNPs without LD around them (Table 2 and Fig. 4), and phenotype-related SNPs with strong LD around them (Table 3 and Fig. 5). This indicated that MBRFS, as a phenotype-related diagrams model fully utilizing conditional independent property, can efficiently capture the true phenotype-related biomarkers no matter whether the phenotype-related biomarkers were independent or correlated, as well as whether the phenotype-related biomarkers were associated with non-phenotype-related ones. More importantly, our proposed MBRFS overcomes the disadvantages of DASSO-MB and FEPI-MB, which would lose their power when numerous phenotype-related SNPs existed on the whole genome and might not work when a phenotype-related SNP located in a high LD block.

In particular, the TDR of MBRFS were always close to zero under null hypothesis (OR = 1 for each SNPs) in all three scenarios (Figs. 3a, 4a and 5a). However, LASSO deviated away from zero for each SNPs under scenario 2 and scenario 3 especially. This suggested that our proposed MBRFS was more stable than other methods. Furthermore, under different OR and MAFs, MBRFS almost had the best performances under three scenarios of independent phenotype-related SNPs without LD around them (Table 1 and Fig. 3b-c), correlated phenotype-related SNPs without LD around them (Table 2 and Fig. 4b-c), and phenotype-related SNPs with strong LD around them (Table 3 and Fig. 5b-c). As expected, when non-significant phenotype-related SNPs emerged under *χ*^2^ test after Bonferroni multiple correction, our proposed MBRFS still detected more phenotype-related SNPs with minor effect, and it had better capability to reduce false negative rate under all the three scenarios (Tables 1, 2 and 3; Figs. 3d, 4d and 5d).

In order to assess the accuracy of MBRFS for capturing the true phenotype-related biomarkers when the correlation existed between phenotype-related and non-phenotype-related biomarkers, simulation scenario 4 with the low, modest and higher LD region (Fig. 6) were performed. The results indicated that MBRFS could exactly identify the true phenotype-related SNPs, while other methods, especially *χ*^2^ test with Bonferroni and B-H adjustment, selected more non-phenotype-related SNPs simultaneously.

In the real data analysis, our proposed MBRFS presented the strongest ability to capture the phenotype-related biomarkers. For Leprosy GWAS data with a total of 491,883 SNPs [22], 13 external validated SNPs were re-identified as Leprosy related SNPs by our proposed MBRFS (see Additional files 6 and 7: Table S1 and Figure S5), and 8 negative SNPs under criteria of Bonferroni adjustment after ATT test were still detected (see Additional file 8: Figure S4). Nevertheless, under the same criteria of positive SNPs clusters, only 7 external validated SNPs were re-identified as positive SNPs by B-H adjustment after ATT test, 5 by Bonferroni adjustment after ATT test, and 3 by LASSO and 1 by DASSO-MB (see Additional files 6 and 7: Table S2 and Figure S5). For DNA methylation and gene expression data, the proposed MBRFS detected relatively reasonable genes, while other methods found too many genes to be interpreted (Additional file 6: Table S3 and S4). For metabolomics dataset, MBRFS identified more verified metabolites, while LASSO method found fewer verified metabolites. The *p* value from logistic regression with Bonferroni and B-H adjustment found four verified metabolites, but they detected too many false positive ones (Additional file 6: Table S5). Of note, for DASSO-MB method, it can only deal with catagorical biomarkers and catagorical phenotype and thus limits its application in above DNA methylation data, gene expression data and metabolomics.

The outstanding performances of our proposed MBRFS can mainly be attribute to increase the power of DASSO-MB [11] and maintain its advantages by modifying its algorithm in three aspects. Firstly, the initially screening procedure by single statistical test not only improves the compute speed, but detects the marginal association between biomarkers and phenotype as many as possible for further conditional independent test in MB algorithm. Secondly, the strategy of the relaxed the conditional independent criterion, reduces the empty cells of the hierarchical contingency table in *G*^2^test when selecting a new biomarker into MB. Thirdly, the proposed repeated-fishing strategy resolves problem of too many empty cells in hierarchical contingency table to maintain the power of *G*^2^ test (Fig. 2). In addition, as our proposed MBRFS stems from causal diagrams and depends on conditional independent test, it has lower false positive rate than LASSO, which is the common drawbacks in various machine leaning algorithms [37, 38]. More importantly, owing to the proposed MBRFS using the repeated fishing strategy, it still captures more phenotype-related SNPs with minor effect and has better capability to reduce false negative rate when non-significant phenotype-related SNPs emerged under *χ*^2^ test after Bonferroni multiple correction.

## Conclusion

Our proposed MBRFS can exactly capture the true phenotype-related biomarkers with the reduction of false negative rate when the phenotype-related biomarkers are independent or correlated, as well as the circumstance that phenotype-related biomarkers are associated with non-phenotype-related ones.

### Patient consent

Obtained informed consent and written.

### Ethics approval

It was approved by ethical committee of Medical Ethical Committee of Qilu Hospital, Shandong University, China.

### Provenance and peer review

Not commissioned; externally peer reviewed.

### Data sharing statement

No additional data are available.

### Availability of supporting data

The gene expression and DNA methylation data for breast cancer can be downloaded from http://cancergenome.nih.gov/publications with application in advance.

## Additional files

Additional file 1: Figure S1. Map phenome (phenotype and various molecular phenotypes) onto genome, including phenotype onto genome (GWAS), transcriptome onto genome (eQTL), proteome onto genome (pQTL), metabolome onto genome (mGWAS), as well as metabolome onto epigenome (mEWAS), epigenome onto genome (meQTL), and phenome onto metabolome (MWAS). (TIF 1116 kb) Additional file 2: Figure S2. The simulation process based on the idea of “gain of function”. (TIF 3829 kb) Additional file 3: Formulas for TDR, FDR and MCC. (DOC 23 kb) Additional file 4: The generation process of independent phenotype-related SNPs. (DOCX 14 kb) Additional file 5: Figure S3. The result of five methods including MBRFS, Bonferroni and B-H adjustment, LASSO, DASSO-MB varying across sample size in former three scenarios. (TIF 153 kb) Additional file 6: Table S1. The identified positive clusters by MBRFS for Leprosy GWAS data. **Table S2.** The identified positive SNPs and clusters by five methods for Leprosy GWAS data. **Table S3.** The identified genes by four methods on breast cancer for DNA methylation data. **Table S4.** The identified genes by four methods on breast cancer for gene expression analysis. **Table S5.** The identified metabolites by four methods on schizophrenia metabolomics data. (DOCX 19 kb) Additional file 7: Figure S5. The found external validated SNPs by five methods in same regions. SNPs marked with red color are the external validated. Black points are the found SNPs by five methods including MBRFS, Bonferroni and B-H adjustment, LASSO, DASSO-MB. (TIF 1107 kb) Additional file 8: Figure S4. The detected 5 SNPs by *χ* ^2^ with Bonferoni adjustment and 8 negative SNPs under Bonferrni adjustment. (TIF 59 kb)

## Abbreviations

MBRFS: Markov Blanket-based repeated-fishing strategy
GWAS: genome-wide association study
SNPs: single nucleotide polymorphisms
LASSO: the least absolute shrinkage and selection operator
LD: linkage disequilibrium
DAG: directed acyclic graph
TDR: true discovery rate
FDR: false discovery rate
MCC: matthews correlation coefficience
MB: Markov Blanket
MAF: minor allele frequency

## References

[1] Haring R, Wallaschofski H (2012). Diving through the “-omics”: the case for deep phenotyping and systems epidemiology. OMICS.

[2] Bender R, Lange S (2001). Adjusting for multiple testing—when and how?. J Clin Epidemiol.

[3] Tian GL, Fang HB, Liu Z, Tan M (2009). Regularized (bridge) logistic regression for variable selection based on ROC criterion. Stat Interface.

[4] Tibshirani R (1996). Regression shrinkage and selection via the lasso. J R Stat Soc Ser B Methodol.

[5] Fan J, Li R (2001). Variable selection via nonconcave penalized likelihood and its oracle properties. J Am Stat Assoc.

[6] Zou H, Hastie T (2005). Regularization and variable selection via the elastic net. J R Stat Soc Ser B (Stat Methodol).

[7] Zou H (2006). The adaptive lasso and its oracle properties. J Am Stat Assoc.

[8] He Q, Lin DY (2011). A variable selection method for genome-wide association studies. Bioinformatics.

[9] Tsamardinos I, Aliferis CF, Statnikov AR (2003). Algorithms for Large Scale Markov Blanket Discovery[C]//FLAIRS Conference 2.

[10] Han B, Park M, Chen XW (2010). A Markov blanket-based method for detecting causal SNPs in GWAS. BMC Bioinformatics.

[11] Han B, Chen XW, Talebizadeh Z (2011). FEPI-MB: identifying SNPs- disease association using a Markov Blanket-based approach. BMC Bioinformatics.

[12] Matthews BW (1975). Comparison of the predicted and observed secondary structure of T4 phage lysozyme. Biochim et Biophys Acta (BBA)- Protein Struct.

[13] Koller D, Sahami M. Toward Optimal Feature Selection[C]//Proc. of International Conference on Machine Learning. [S. l.]: Morgan Kaufmann Publishers. 1996;284–292.

[14] Margaritis D., Thrun, S. Bayesian network induction via local neighborhoods [C] II Advances in Neural Information Processing Systems. 1999; 505–511.

[15] Zhang Y, Zhang Z, Liu K, Qian G (2010). An improved IAMB algorithm for Markov blanket discovery. J Comput.

[16] Aliferis C F, Tsamardinos I, Statnikov A. HITON: a novel Markov Blanket algorithm for optimal variable selection[C]//AMIA Annual Symposium Proceedings. Am Med Inform Assoc. 2003;21:5. PMC1480117 14728126

[17] Westfall PH, Johnson WO, Utts JM (1997). A Bayesian perspective on the Bonferroni adjustment. Biometrika.

[18] Reiner A, Yekutieli D, Benjamini Y (2003). Identifying differentially expressed genes using false discovery rate controlling procedures. Bioinformatics.

[19] Li J, Chen Y (2008). Generating samples for association studies based on HapMap data. BMC Bioinformatics.

[20] Gaiano N, Kohtz JD, Turnbull DH, Fishell G (1999). A method for rapid gain-of-function studies in the mouse embryonic nervous system. Nat Neurosci.

[21] Winklhofer KF, Tatzelt J, Haass C (2008). The two faces of protein misfolding: gain-and loss-of-function in neurodegenerative diseases. EMBO J.

[22] Zhang FR, Huang W, Chen SM, Sun LD, Liu H, Li Y (2009). Genomewide association study of leprosy. N Engl J Med.

[23] Weinstein JN, Collisson EA, Mills GB, Shaw KR, Cancer Genome Atlas Research N (2013). The Cancer Genome Atlas Pan-Cancer analysis project. Nat Genet.

[24] Antoniou AC, Kuchenbaecker KB, Soucy P (2012). Common variants at 12p11, 12q24, 9p21, 9q31. 2 and in ZNF365 are associated with breast cancer risk for BRCA1 and/or BRCA2 mutation carriers[J]. Breast Cancer Res.

[25] Chioni AM, Brackenbury WJ, Calhoun JD (2009). A novel adhesion molecule in human breast cancer cells: Voltage-gated Na + channel β1 subunit[J]. Int J Biochem Cell Biol.

[26] Wang JL, Tong CW, Chang WT (2013). Novel genes FAM134C, C3orf10 and ENOX1 are regulated by NRF-1 and differentially regulate neurite outgrowth in neuroblastoma cells and hippocampal neurons[J]. Gene.

[27] McPherson JR, Ong CK, Ng CCY (2015). Whole-exome sequencing of breast cancer, malignant peripheral nerve sheath tumor and neurofibroma from a patient with neurofibromatosis type 1[J]. Cancer Med.

[28] Mosca E, Alfieri R, Merelli I (2010). A multilevel data integration resource for breast cancer study[J]. BMC Syst Biol.

[29] Jiao K, Zhou Y, Hogan BL (2002). Identification of mZnf8, a mouse Krüppel-like transcriptional repressor, as a novel nuclear interaction partner of Smad1. Mol Cell Biol.

[30] Xu K, Cui J, Olman V (2010). A comparative analysis of gene-expression data of multiple cancer types[J]. PLoS One.

[31] Dago DN, Scafoglio C, Rinaldi A (2015). Estrogen receptor beta impacts hormone-induced alternative mRNA splicing in breast cancer cells[J]. BMC Genomics.

[32] Radke I, Götte M, Kersting C (2006). Expression and prognostic impact of the protein tyrosine phosphatases PRL-1, PRL-2, and PRL-3 in breast cancer[J]. Br J Cancer.

[33] Daniels MJ, Wang Y, Lee M, Venkitaraman AR (2004). Abnormal cytokinesis in cells deficient in the breast cancer susceptibility protein BRCA2. Science.

[34] Karpinski P, Ramsey D, Grzebieniak Z, Sasiadek MM, Blin N (2008). The CpG island methylator phenotype correlates with long-range epigenetic silencing in colorectal cancer. Mol Cancer Res.

[35] Kichine E, Rozé V, Mitchell MJ (2012). HSFY genes and the P4 palindrome in the AZFb interval of the human Y chromosome are not required for spermatocyte maturation. Hum Reprod.

[36] Liu Y, Zhang T, Wang L, Liu J, Chang X, Zhang J (2015). Serum metabolic profiling of schizophrenia based on random forest. J Shandong Univ (Health Sci).

[37] Ritchie MD, Hahn LW, Moore JH (2003). Power of multifactor dimensionality reduction for detecting gene-gene interactions in the presence of genotyping error, missing data, phenocopy, and genetic heterogeneity. Genet Epidemiol.

[38] Zhang Y, Liu JS (2007). Bayesian inference of epistatic interactions in case–control studies. Nat Genet.

