# Supplementary figures and images for: A novel Markov Blanket-based repeated-fishing strategy for capturing phenotype-related biomarkers in big omics data
Source: BMC Genet. 2016 Mar 9;17:51. doi: 10.1186/s12863-016-0358-5 (PMC4784463; doi:10.1186/s12863-016-0358-5)

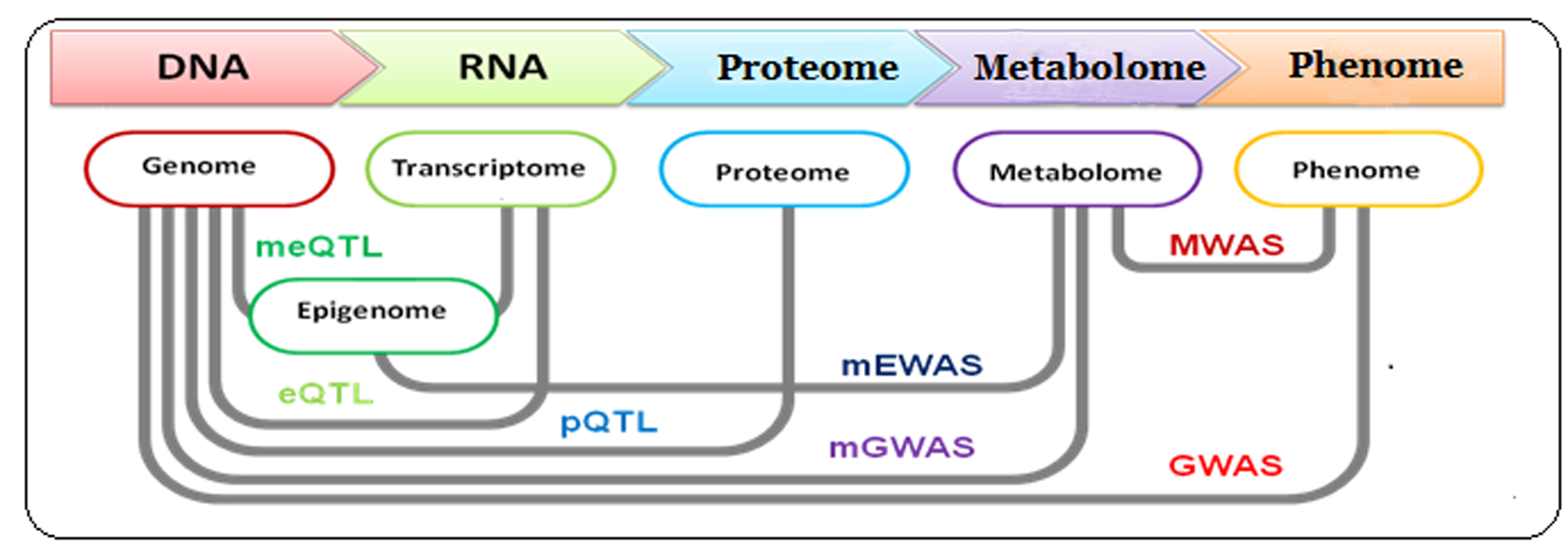

Supplement: Additional file 1: Figure S1. — Map phenome (phenotype and various molecular phenotypes) onto genome, including phenotype onto genome (GWAS), transcriptome onto genome (eQTL), proteome onto genome (pQTL), metabolome onto genome (mGWAS), as well as metabolome onto epigenome (mEWAS), epigenome onto genome (meQTL), and phenome onto metabolome (MWAS). (TIF 1116 kb) [file 12863_2016_358_MOESM1_ESM.tif]

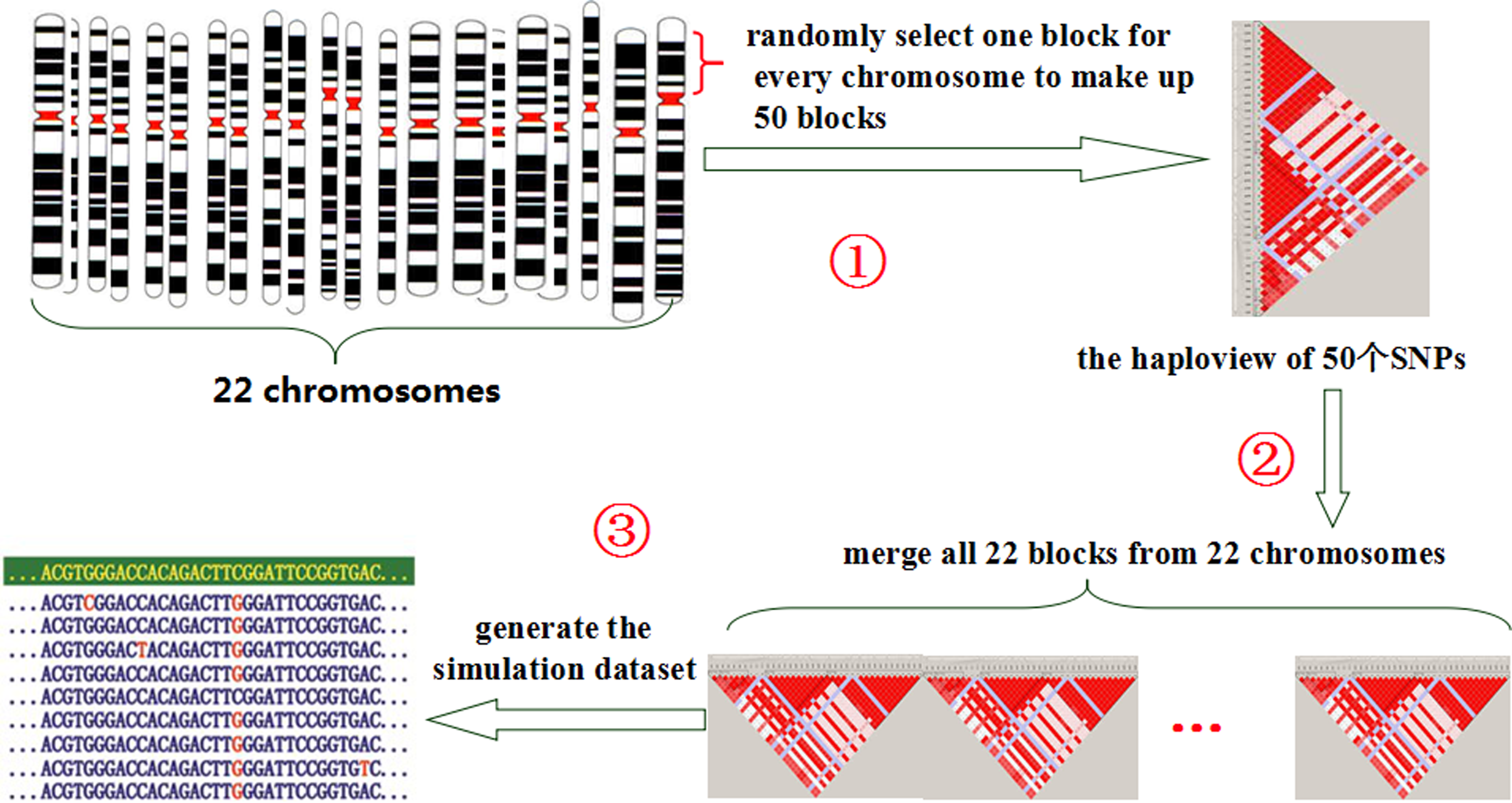

Supplement: Additional file 2: Figure S2. — The simulation process based on the idea of “gain of function”. (TIF 3829 kb) [file 12863_2016_358_MOESM2_ESM.tif]

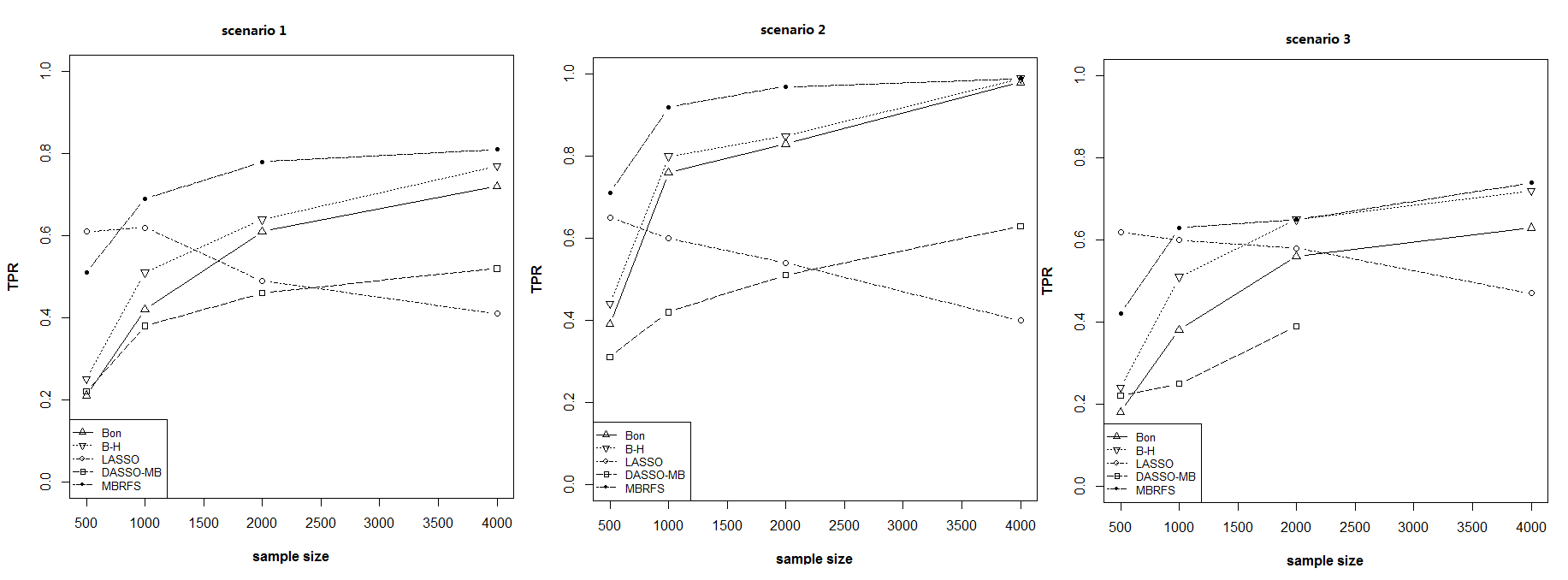

Supplement: Additional file 5: Figure S3. — The result of five methods including MBRFS, Bonferroni and B-H adjustment, LASSO, DASSO-MB varying across sample size in former three scenarios. (TIF 153 kb) [file 12863_2016_358_MOESM5_ESM.tif]

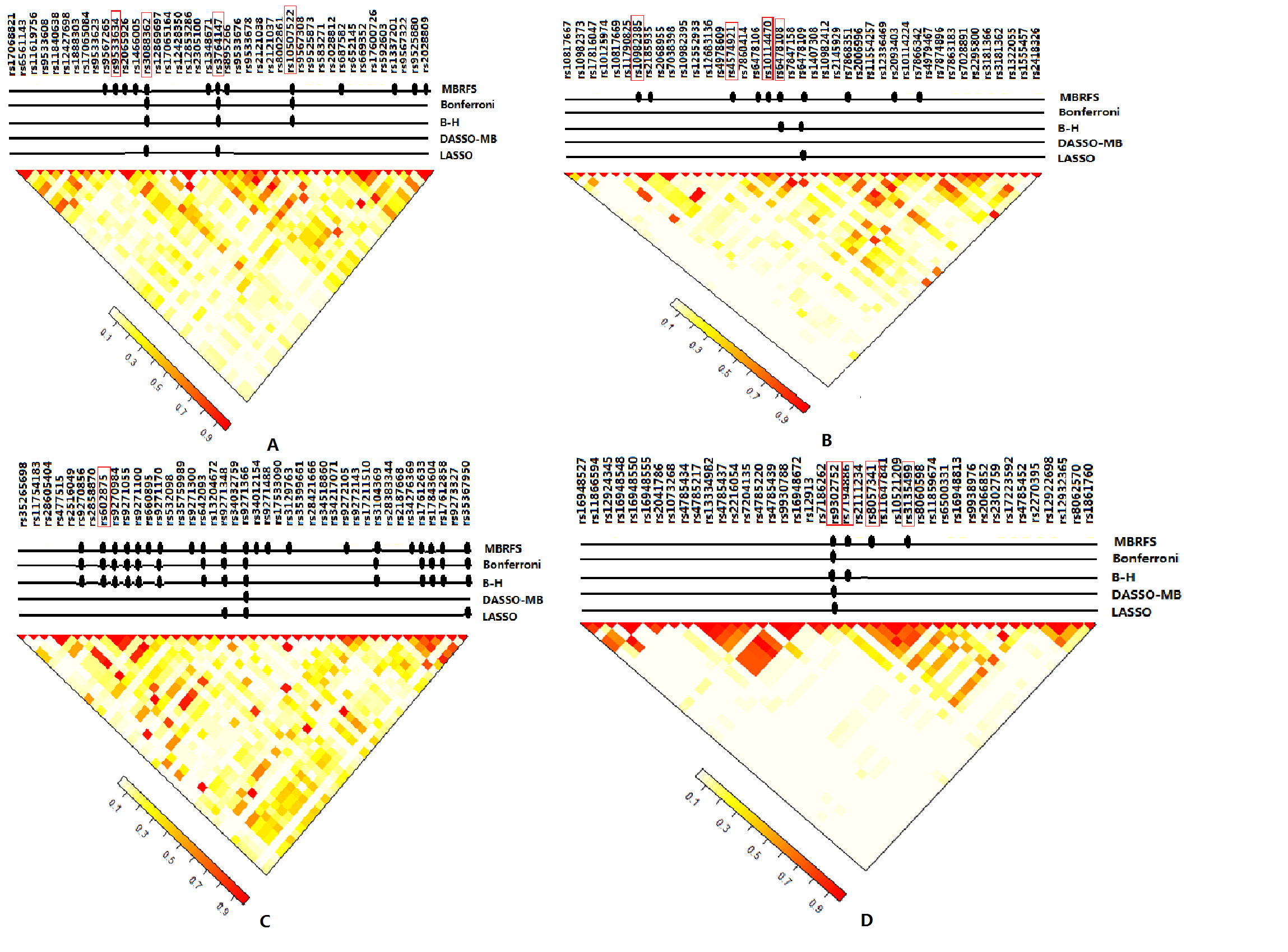

Supplement: Additional file 7: Figure S5. — The found external validated SNPs by five methods in same regions. SNPs marked with red color are the external validated. Black points are the found SNPs by five methods including MBRFS, Bonferroni and B-H adjustment, LASSO, DASSO-MB. (TIF 1107 kb) [file 12863_2016_358_MOESM7_ESM.tif]

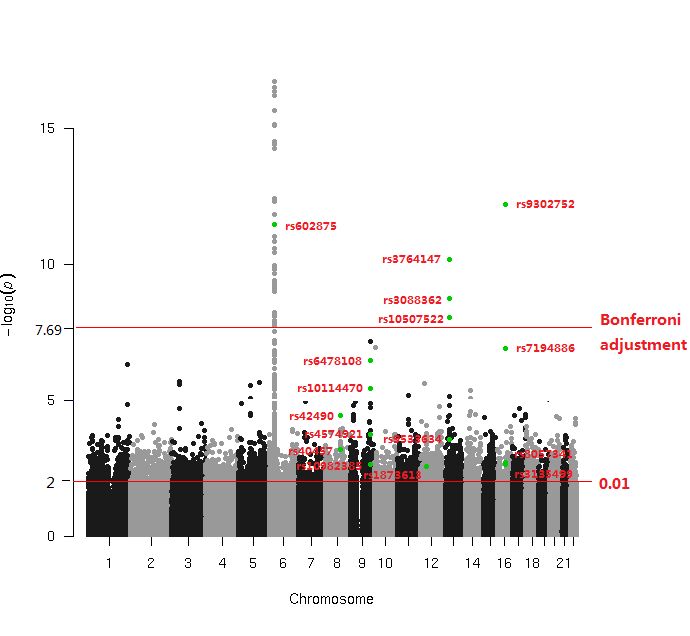

Supplement: Additional file 8: Figure S4. — The detected 5 SNPs by χ 2 with Bonferoni adjustment and 8 negative SNPs under Bonferrni adjustment. (TIF 59 kb) [file 12863_2016_358_MOESM8_ESM.tif]
