# Supplementary material for: A novel Markov Blanket-based repeated-fishing strategy for capturing phenotype-related biomarkers in big omics data
Source: BMC Genet. 2016 Mar 9;17:51. doi: 10.1186/s12863-016-0358-5 (PMC4784463; doi:10.1186/s12863-016-0358-5)
Supplement: Additional file 3: — Formulas for TDR, FDR and MCC. (DOC 23 kb) [file 12863_2016_358_MOESM3_ESM.doc]

**1. Formulas for TDR, FDR and MCC:**

To assess the performances of compared methods (e.g. MBRFS, LASSO, DASSO-MB, etc. in our manuscript), two indexes, false positive (using FDR), false negative (using TDR), must usually be reported. The false positive is an error in outcome reporting in which biomarkers are selected out by a specific method from the simulation data when they are not previously defined as causal biomarkers, while a false negative is an error in which the previously defined causal biomarkers were not selected out from the simulation data. Ideally, a good method should not only hold low false positive rate (FDR) but low false negative rate (i.e. high TDR). Nevertheless, false positive and false negative usually reveal opposite trends, sometimes, it is difficult to get their low level simultaneously for a specific method. Therefore, to evaluate and compare the performances of different methods objectively and reasonably, a combined index of FDR and TDR (i.e. MCC) had been created for trading off between false positive and false negative in 1975, and it was widely used to assess the performances of different models in machine learning, and in omics data analysis nowadays. A good method should hold a high MCC value, when false positive and false negative have an opposite trend.

In our study, FDR, TDR and MCC were separately defined as

$$\begin{matrix} \text{TDR}=\frac{\text{nu}mber of positive SNPs with OR\neq1}{\text{nu}mber of \text{sp}ecified causal SNPs } \\ FDR=\frac{\text{nu}mber of positive SNPs with OR=1}{\text{nu}mber of positive SNPs } \\ MCC=\frac{TP\times TN-FP\times FN}{\sqrt{(TP+FP)(TP+FN)(TN+FP)(TN+FN)}} \end{matrix}$$

where, TP is the number of [true positives](https://en.wikipedia.org/wiki/True_positive), TN the number of [true negatives](https://en.wikipedia.org/wiki/True_negative), FP the number of [false positives](https://en.wikipedia.org/wiki/False_positive) and FN the number of [false negatives](https://en.wikipedia.org/wiki/False_negative). If any of the four sums in the denominator is zero, the denominator can be arbitrarily set to one; this results in a Matthews correlation coefficient of zero, which can be shown to be the correct limiting value.
