# Supplementary material for: A novel Markov Blanket-based repeated-fishing strategy for capturing phenotype-related biomarkers in big omics data
Source: BMC Genet. 2016 Mar 9;17:51. doi: 10.1186/s12863-016-0358-5 (PMC4784463; doi:10.1186/s12863-016-0358-5)
Supplement: Additional file 4: — The generation process of independent phenotype-related SNPs. (DOCX 14 kb) [file 12863_2016_358_MOESM4_ESM.docx]

**2. The 8 independent and correlated phenotype-related SNPs were generated by logistic model**

First, a uniform distribution was generated, cutting this distribution into three groups (i.e. 0, 1, 2) based on the given MAF. similarly, 8 correlated SNPs were generated by multiple normal distribution with correlation correlation coefficient 0.1 then cutting this distribution into three groups (i.e. 0, 1, 2) based on the given MAF. Above process was repeated independently to generate 8 independent or correlated phenotype-related SNPs. Phenotype $Y$ was generated based on logistic model,

$p(Y=1)=\frac{\exp(\alpha+\beta_{1}SNP_{1}+\beta_{2}SNP_{2}+\beta_{3}SNP_{3}+\beta_{4}SNP_{4}+\beta_{5}SNP_{5}+\beta_{6}SNP_{6}+\beta_{7}SNP_{7}+\beta_{8}SNP_{8})}{1+\exp(\alpha+\beta_{1}SNP_{1}+\beta_{2}SNP_{2}+\beta_{3}SNP_{3}+\beta_{4}SNP_{4}+\beta_{5}SNP_{5}+\beta_{6}SNP_{6}+\beta_{7}SNP_{7}+\beta_{8}SNP_{8})}$Finally, Y(0,1) were generated based on binomial distribution based on the probability.
