# Supplementary material for: A novel Markov Blanket-based repeated-fishing strategy for capturing phenotype-related biomarkers in big omics data
Source: BMC Genet. 2016 Mar 9;17:51. doi: 10.1186/s12863-016-0358-5 (PMC4784463; doi:10.1186/s12863-016-0358-5)
Supplement: Additional file 6: Table S1. — The identified positive clusters by MBRFS for Leprosy GWAS data. Table S2. The identified positive SNPs and clusters by five methods for Leprosy GWAS data. Table S3. The identified genes by four methods on breast cancer for DNA methylation data. Table S4. The identified genes by four methods on breast cancer for gene expression analysis. Table S5. The identified metabolites by four methods on schizophrenia metabolomics data. (DOCX 19 kb) [file 12863_2016_358_MOESM6_ESM.docx]

**Table S1. The identified positive clusters by MBRFS for Leprosy GWAS data**

| **Cluster** | **SNPs in positive cluster** | **Position range (bp)** | **Average R^2^** | **Chromosome** |
| --- | --- | --- | --- | --- |
| 1 | rs9270856 rs602875 rs9270984 rs9271055 rs9271100 rs660895 rs9271170 rs9271300 rs642093 rs9271348 rs9271366 rs34012154 rs9271488 rs3129763 rs9272105 rs3104369 rs34276369 rs17612633 rs17843604 rs17612858 rs35367950 | 32678817-  32732638 | 0.38 | 6 |
| 2 | rs9302752 rs7194886 rs8057341 rs3135499 | 49276604-  49323628 | 0.21 | 16 |
| 3 | rs9567265 rs9533634 rs2065926 rs1466005 rs3088362 rs1348671 rs3764147 rs895266 rs10507522 rs687582 rs1337201 rs9525880 rs2028809 | 43284266-  43421136 | 0.23 | 13 |
| 4 | rs10982385 rs2185935 rs4574921 rs6478106 rs10114470 rs6478108 rs6478109 rs7868351 rs2093403 rs7866342 | 116532838-  116667390 | 0.30 | 9 |
| 5 | rs12878420 rs12436400 rs10150753 rs10133203 rs4898725 rs3742539 rs3825597 | 51406432-  51488595 | 0.21 | 14 |
| 6 | rs9268542 rs3135363 rs9268585 rs9268589 rs9501626 rs3135338 rs2027856 rs9268615 rs2395173 rs3129871 rs3129882 rs6937545 | 32492699-  32526009 | 0.29 | 6 |
| 7 | rs656111 | 9978931 | NA | 20 |
| 8 | rs2491231 rs2491236 rs6491248 rs1933437 | 27508183-  27522294 | 0.53 | 13 |
| 9 | rs3935248 rs7633016 rs4076927 rs7650366 rs9815362 rs6808303 rs6793661 rs6763438 rs7614762 rs6787229 | 46688869-  46864191 | 0.34 | 3 |
| 10 | rs2856695 rs17212420 rs4538747 rs9275224 rs9275245 rs5000634 rs3129720 rs6457617 rs2647012 rs9357152 rs9275312 rs2856725 rs9275328 rs2858312 rs2647050 rs2856718 rs2856717 rs2858305 | 32759872-  32778442 | 0.32 | 6 |
| 11 | rs498972 rs672717 | 41836890-  41844065 | 0.23 | 18 |
| 12 | rs9918306 | 30527750 | NA | 6 |
| 13 | rs2844623 rs7759127 rs2524077 rs2524073 rs2524070 rs2524068 rs2524057 | 31340522-  31359874 | 0.36 | 6 |
| 14 | rs1390568 rs6863200 rs2029889 rs1496286 rs12153172 rs7730186 rs4866111 rs1390566 rs1845830 rs393268 | 18607264-  18730232 | 0.36 | 5 |
| 15 | rs4310466 | 106848310 | NA | 1 |
| 16 | rs989817 rs2913319 rs11949165 rs11958839 rs2913326 rs6874946 rs17106076 | 146472467-  146531582 | 0.30 | 5 |
| 17 | rs2844649 | 31012251 | NA | 6 |
| 18 | rs741437 rs6734024 | 15543778-  15635631 | 0.46 | 2 |

**Table S2. The identified positive SNPs and clusters by five methods for Leprosy GWAS data**

| **Methods** | **Number of identified external validated SNPs** | **Number of identified all positive SNPs** | **Number of identified all positive SNPs clusters** |
| --- | --- | --- | --- |
| MBRFS | 13 | 128 | 18 |
| ATT with Bonferroni adjustment | 5 | 45 | 9 |
| ATT with B-H adjustment | 7 | 72 | 20 |
| DASSO-MB | 1 | 2 | 2 |
| LASSO | 3 | 8 | 5 |

**Table S3. The identified genes by four methods on breast cancer for DNA methylation data**

| **Methods** | **The discovered markers** |
| --- | --- |
| Logistic regression with Bonferroni adjustment | 0 |
| Logistic regression with B-H adjustment | 0 |
| LASSO | SNX19 MAPK1IP1L ANAPC2.SSNA1 DBF4.SLC25A40  STXBP5.1 CPEB2 ATP9A SNRPE  HSD17B7.1 ZNF800 ERP44.INVS EIF3J  CKAP2.1 ATG4D UBR3 DNAJC5B  CCDC120.1 NA..981 SLC11A2 LOC285954.INHBA  KLF10.1 DCAF4.1 CXCL13 HDAC8  NA..1122 ERICH1.1 NA..1378 EIF3I.C1orf91.2  SCN4B.1 |
| MBRFS | CCDC91 SCN4B.1 C3orf10 |

**Table S4. The identified genes by four methods on breast cancer for gene expression analysis**

| **Methods** | **The discovered markers** |
| --- | --- |
| Logistic regression with Bonferroni adjustment | 0 |
| Logistic regression B-H adjustment | 0 |
| LASSO | FAM71C |
| MBRFS | NRXN1 FAM71C ZNF8 FGL1 ZNF438 PTP4A3 INCENP  SCTR CYorf15A |

**Table S5. The identified metabolites by four methods on** [**schizophrenia**](javascript:void(0);) **metabolomics data**

| **Methods** | **Number of verified positive biomarkers** | **Number of not verified positive biomarkers** | **Total number of found biomarkers** |
| --- | --- | --- | --- |
| Logistic regression with Bonferroni adjustment | 4 | 222 | 226 |
| Logistic regression B-H adjustment | 4 | 818 | 822 |
| LASSO | 1 | 52 | 53 |
| MBRFS | 3 | 57 | 60 |
